# The genome sequence of the Early Bumblebee, *Bombus pratorum* (Linnaeus, 1761)

**DOI:** 10.12688/wellcomeopenres.19250.1

**Published:** 2023-03-29

**Authors:** Liam M. Crowley, Olga Sivell, Duncan Sivell, University of Oxford and Wytham Woods Genome Acquisition Lab, Natural History Museum Genome Acquisition Lab, Darwin Tree of Life Barcoding collective, Wellcome Sanger Institute Tree of Life programme, Wellcome Sanger Institute Scientific Operations: DNA Pipelines collective, Tree of Life Core Informatics collective, Darwin Tree of Life Consortium

**Affiliations:** 1 University of Oxford, Oxford, England, UK; 2 Natural History Museum, London, England, UK

**Keywords:** Bombus pratorum, Early Bumblebee, genome sequence, chromosomal, Hymenoptera

## Abstract

We present a genome assembly from an individual female *Bombus pratorum* (the Early Bumblebee; Arthropoda; Insecta; Hymenoptera; Apidae). The genome sequence is 285.1 megabases in span. Most of the assembly is scaffolded into 18 chromosomal pseudomolecules. The mitochondrial genome has also been assembled and is 21.5 kilobases in length. Gene annotation of this assembly on Ensembl identified 13,746 protein coding genes.

## Species taxonomy

Eukaryota; Metazoa; Ecdysozoa; Arthropoda; Hexapoda; Insecta; Pterygota; Neoptera; Endopterygota; Hymenoptera; Apocrita; Aculeata; Apoidea; Apidae; *Bombus*; *Pyrobombus*; *Bombus pratorum* (Linnaeus, 1761) (NCBI:txid30194).

## Background

The Early Bumblebee, *Bombus pratorum*, is one of the seven most common and widespread species of bumblebees in the UK. It is generally common throughout its range, that includes much of Europe across to the near East. It can be found in many different habitats, predominantly gardens and woodlands. It has been shown that the abundance of this species exhibits a positive correlation to the degree of gardens and allotments in the landscape ( Foster *et al.*, 2017). It is a small bumblebee species less than 17 mm, covered in black hairs with bands of yellow hairs across the pronotum and second tergite, and red hairs on the apical segments of the abdomen. It is a eusocial species with reproductive queens and males, and non-reproductive workers. Males have yellow hairs on the head and face.

It typically has the earliest appearing workers of any UK bumblebee, with workers of the first brood appearing from as early as February ( Benton, 2006). *Bombus pratorum* is frequently bivoltine in the UK, particularly in the south, becoming increasingly univoltine further northwards ( Prŷs-Jones & Corbet, 1987). Males and new queens from the first colony cycle can be produced from May and June respectively. The colony cycle is remarkably short, with reproductives being produced in as little as 3 months after founding ( Edwards & Jenner, 2005) and new queens being one of the first UK bumblebee species to enter overwintering diapause ( Alford, 1969). Nests are constructed in a variety of situations, both above- and below-ground, including in old small-mammal burrows and aerial cavities such as bird boxes and holes in trees ( Lye *et al.*, 2012). Colonies are relatively small, usually peaking at fewer than 100 workers. In UK agricultural landscapes, nest density is estimated at 26 nests per square kilometre, and minimum estimated maximum foraging range is 674 m ( Knight *et al.*, 2005).

It is polylectic, although a preference for pollen from Fabaceae has been found ( Goulson & Darvill, 2004). It visits a wide range of flowers for nectar, particularly favouring Blackthorn ( *Prunus spinosa*), Bramble ( *Rubus fruticosus* agg.) and Raspberry ( *Rubus idaeus*), and is an important pollinator of soft fruits.

A complete genome sequence for this species will facilitate studies into the evolution of eusociality, conservation of important pollinator species, reproductive evolution and foraging behaviour.

## Genome sequence report

**Figure 1.**
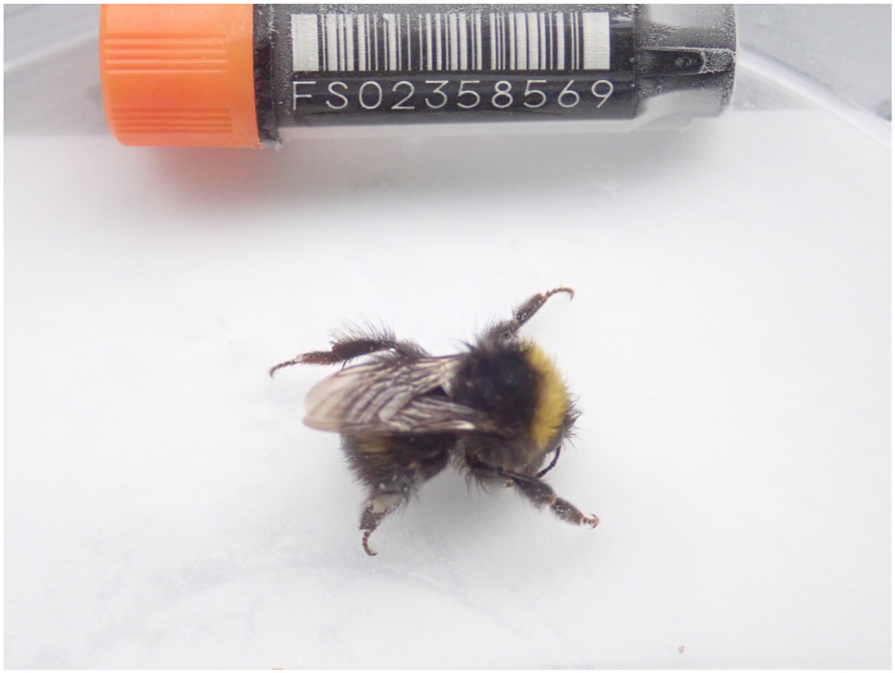
Photograph of the *Bombus pratorum* (iyBomPrat1) specimen used for genome sequencing.

The genome was sequenced from one female *Bombus pratorum* specimen ( Figure 1) collected from Wytham Woods, Oxfordshire, UK (latitude 51.77, longitude –1.33). A total of 60-fold coverage in Pacific Biosciences single-molecule HiFi long reads and 88-fold coverage in 10X Genomics read clouds were generated. Primary assembly contigs were scaffolded with chromosome conformation Hi-C data. Manual assembly curation corrected 32 missing joins or mis-joins, reducing the scaffold number by 24.24%, and increasing the scaffold N50 by 20.98%.

**Figure 2.**
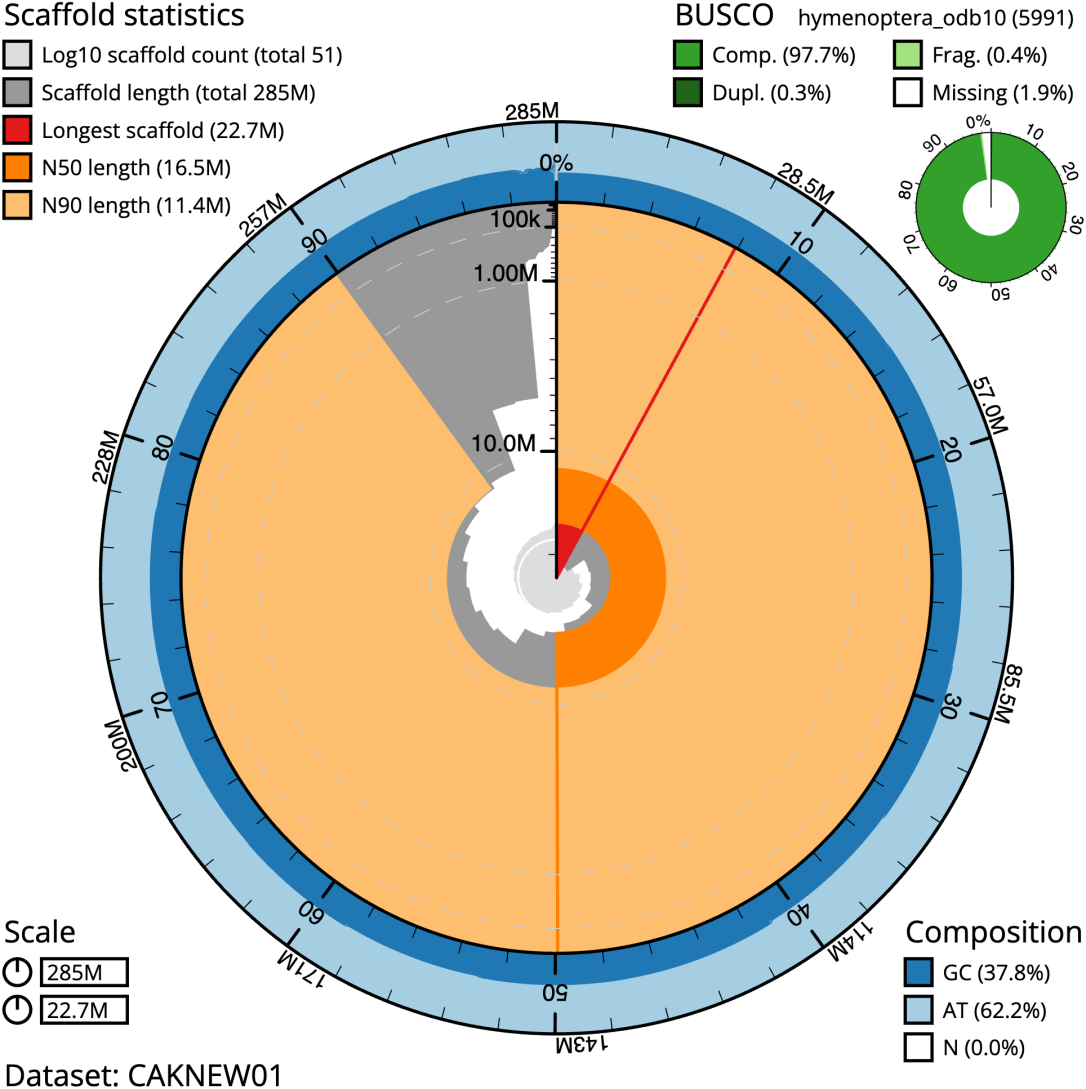
Genome assembly of *Bombus pratorum*, iyBomPrat1.1: metrics. The BlobToolKit Snailplot shows N50 metrics and BUSCO gene completeness. The main plot is divided into 1,000 size-ordered bins around the circumference with each bin representing 0.1% of the 285,072,354 bp assembly. The distribution of scaffold lengths is shown in dark grey with the plot radius scaled to the longest scaffold present in the assembly (22,727,794 bp, shown in red). Orange and pale-orange arcs show the N50 and N90 scaffold lengths (16,494,161 and 11,389,935 bp), respectively. The pale grey spiral shows the cumulative scaffold count on a log scale with white scale lines showing successive orders of magnitude. The blue and pale-blue area around the outside of the plot shows the distribution of GC, AT and N percentages in the same bins as the inner plot. A summary of complete, fragmented, duplicated and missing BUSCO genes in the hymenoptera_odb10 set is shown in the top right. An interactive version of this figure is available at https://blobtoolkit.genomehubs.org/view/iyBomPrat1.1/dataset/CAKNEW01/snail.

The final assembly has a total length of 285.1 Mb in 50 sequence scaffolds with a scaffold N50 of 16.5 Mb ( Table 1). Most (96.23%) of the assembly sequence was assigned to 18 chromosomal-level scaffolds. Chromosome-scale scaffolds confirmed by the Hi-C data are named in order of size ( Figure 2– Figure 5; Table 2). While not fully phased, the assembly deposited is of one haplotype. Contigs corresponding to the second haplotype have also been deposited.

**Table 1.** Genome data for *Bombus pratorum*, iyBomPrat1.1.

Project accession data		
Assembly identifier	iyBomPrat1.1	
Species	*Bombus pratorum*	
Specimen	iyBomPrat1	
NCBI taxonomy ID	30194	
BioProject	PRJEB48116	
BioSample ID	SAMEA7520485	
Isolate information	iyBomPrat1, female, head and thorax (genome sequencing); abdomen (Hi-C) iyBomPrat2, female (RNA sequencing)	
Assembly metrics *		*Benchmark*
Consensus quality (QV)	57.3	*≥ 50*
*k*-mer completeness	99.99%	*≥ 95%*
BUSCO **	C:97.7%[S:97.4%,D:0.3%], F:0.4%,M:1.9%,n:5,991	*C ≥ 95%*
Percentage of assembly mapped to chromosomes	96.23%	*≥ 95%*
Sex chromosomes	Not applicable	*localised homologous pairs*
Organelles	Mitochondrial genome assembled	*complete single alleles*
Raw data accessions		
PacificBiosciences SEQUEL II	ERR7123979	
10X Genomics Illumina	ERR7113571–ERR7113574	
Hi-C Illumina	ERR7113575	
PolyA RNA-Seq Illumina	ERR7113576, ERR7113577	
Genome assembly		
Assembly accession	GCA_930367275.1	
*Accession of alternate haplotype*	GCA_930367225.1	
Span (Mb)	285.1	
Number of contigs	90	
Contig N50 length (Mb)	8.5	
Number of scaffolds	50	
Scaffold N50 length (Mb)	16.5	
Longest scaffold (Mb)	22.7	
**Genome annotation**		
Number of protein-coding genes	13,746	
Number of non-coding genes	5,678	
Number of gene transcripts	40,821	

* Assembly metric benchmarks are adapted from column VGP-2020 of “Table 1: Proposed standards and metrics for defining genome assembly quality” from ( Rhie *et al.*, 2021). ** BUSCO scores based on the hymenoptera_odb10 BUSCO set using v5.3.2. C = complete [S = single copy, D = duplicated], F = fragmented, M = missing, n = number of orthologues in comparison. A full set of BUSCO scores is available at https://blobtoolkit.genomehubs.org/view/iyBomPrat1.1/dataset/CAKNEW01/busco.

**Figure 3.**
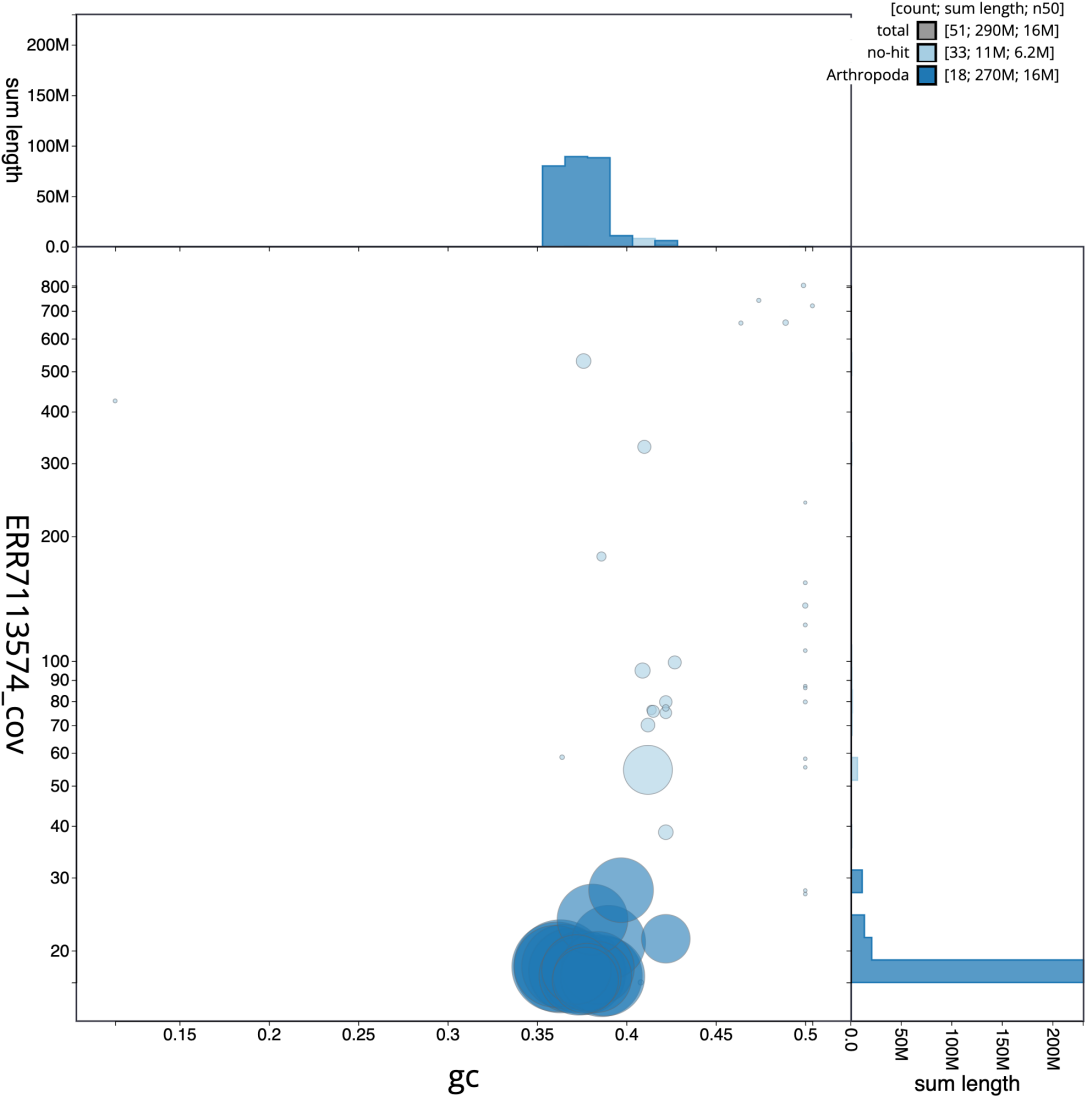
Genome assembly of *Bombus pratorum*, iyBomPrat1.1: GC coverage. BlobToolKit GC-coverage plot. Scaffolds are coloured by phylum. Circles are sized in proportion to scaffold length. Histograms show the distribution of scaffold length sum along each axis. An interactive version of this figure is available at https://blobtoolkit.genomehubs.org/view/iyBomPrat1.1/dataset/CAKNEW01/blob.

**Figure 4.**
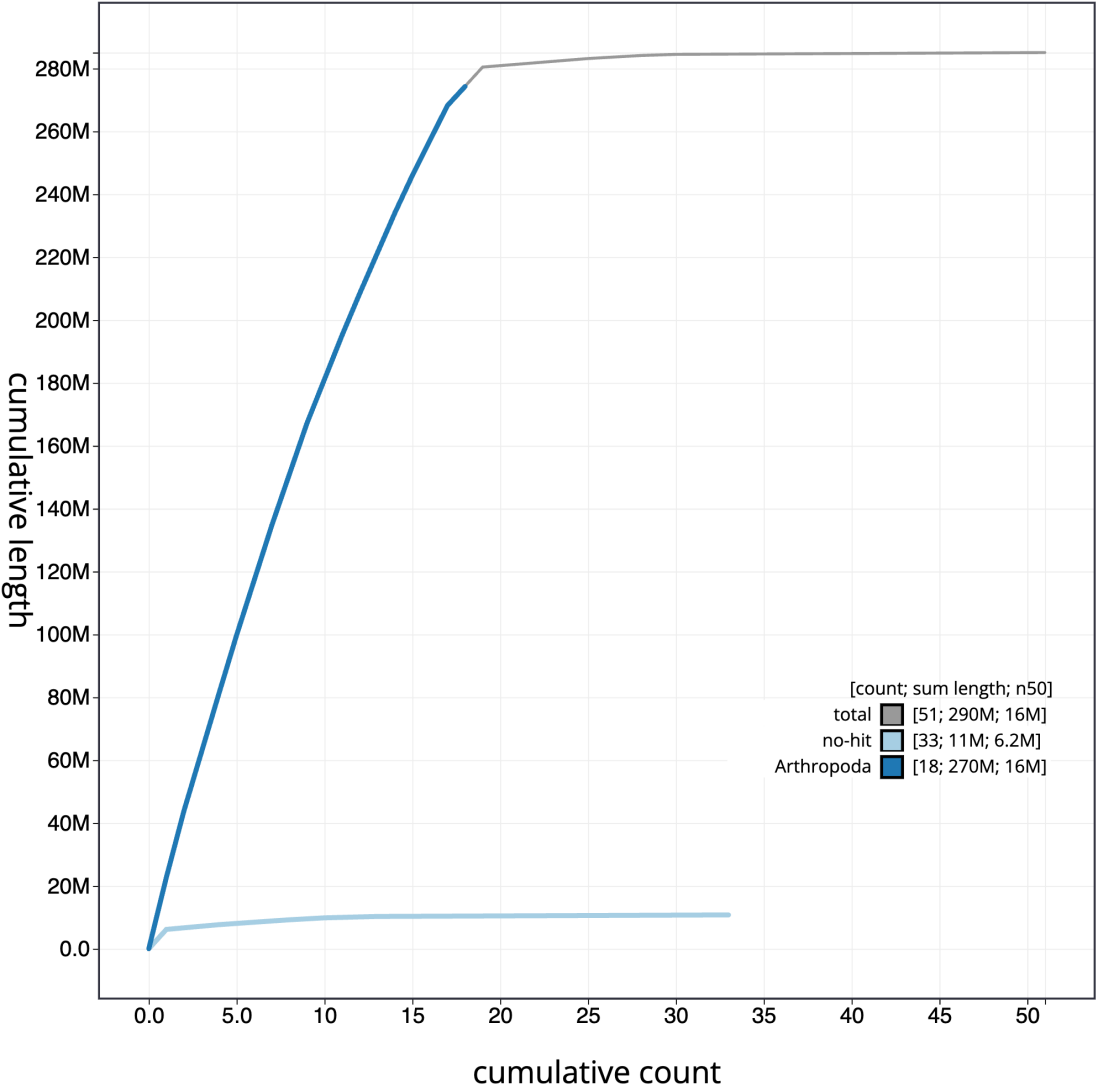
Genome assembly of *Bombus pratorum*, iyBomPrat1.1: cumulative sequence. BlobToolKit cumulative sequence plot. The grey line shows cumulative length for all scaffolds. Coloured lines show cumulative lengths of scaffolds assigned to each phylum using the buscogenes taxrule. An interactive version of this figure is available at https://blobtoolkit.genomehubs.org/view/iyBomPrat1.1/dataset/CAKNEW01/cumulative.

**Figure 5.**
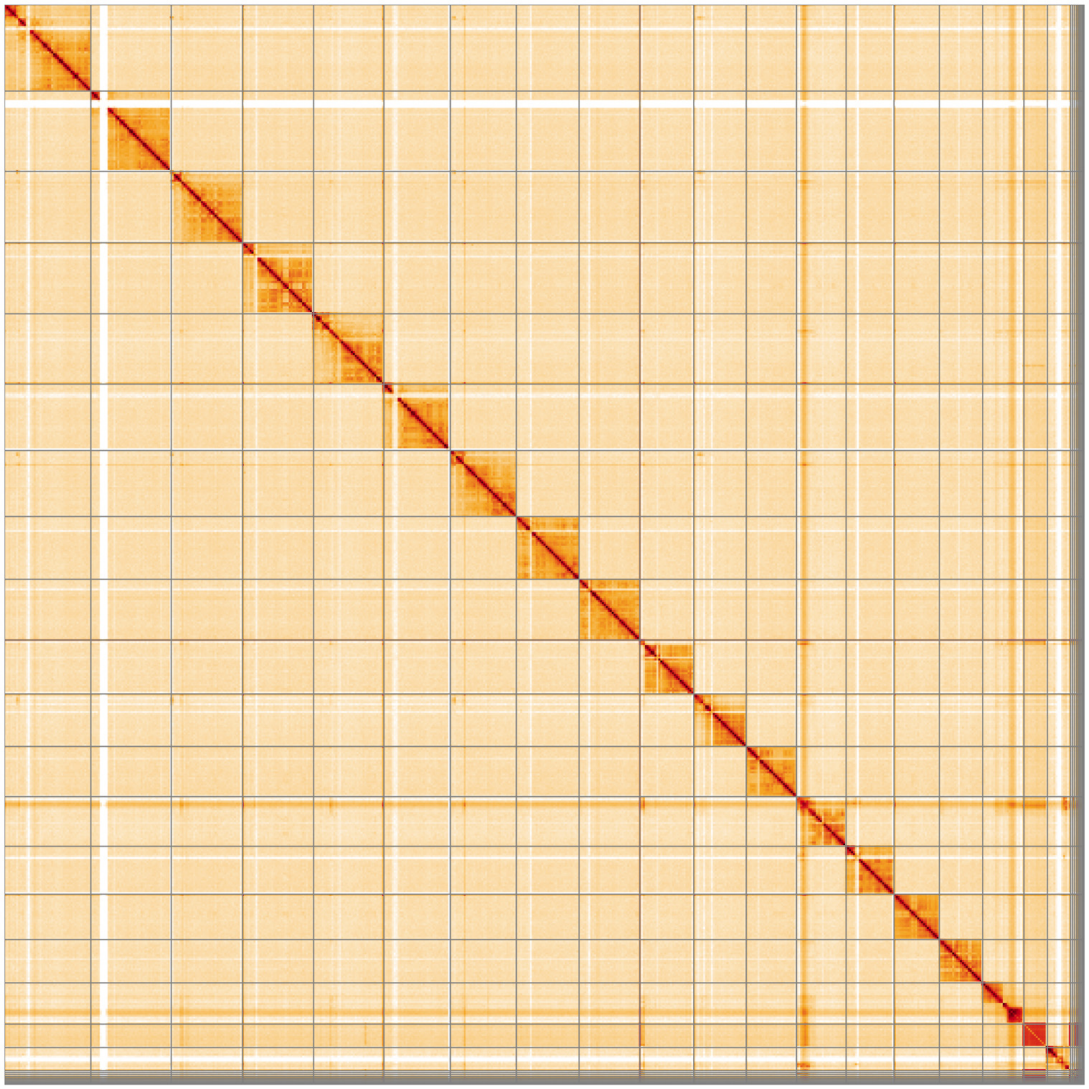
Genome assembly of *Bombus pratorum*, iyBomPrat1.1: Hi-C contact map. Hi-C contact map of the iyBomPrat1.1 assembly, visualised using HiGlass. Chromosomes are shown in order of size from left to right and top to bottom. An interactive version of this figure may be viewed at https://genome-note-higlass.tol.sanger.ac.uk/l/?d=X2H_v2CFRT2Nc-jNlyp_Tg.

**Table 2.** Chromosomal pseudomolecules in the genome assembly of *Bombus pratorum*, iyBomPrat1.

INSDC accession	Chromosome	Size (Mb)	GC%
OV883983.1	1	22.73	36.3
OV883984.1	2	21.13	36.1
OV883985.1	3	18.82	36.9
OV883986.1	4	18.65	36.5
OV883987.1	5	18.5	37.6
OV883988.1	6	17.49	36
OV883989.1	7	17.37	38.6
OV883990.1	8	16.49	38.8
OV883991.1	9	15.97	37.3
OV883992.1	10	14.22	39
OV883993.1	11	13.8	38.4
OV883994.1	12	13.24	38.3
OV883995.1	13	13.03	38.1
OV883996.1	14	12.57	37.2
OV883997.1	15	11.97	37.8
OV883998.1	16	11.39	37.7
OV883999.1	17	10.86	39.7
OV884000.1	18	6.06	42.2
OV884001.1	MT	0.02	11.3
-	unplaced	10.76	41.4

The estimated Quality Value (QV) of the final assembly is 57.3 with *k*-mer completeness of 99.99%, and the assembly has a BUSCO v5.3.2 ( Manni *et al.*, 2021) completeness of 97.7% (single 97.4%, duplicated 0.3%) using the hymenoptera_odb10 reference set ( *n* = 5,991).

## Genome annotation report

The *B. pratorum* genome assembly GCA_930367275.1 was annotated using the Ensembl rapid annotation pipeline ( Table 1; https://rapid.ensembl.org/Bombus_pratorum_GCA_930367275.1/Info/Index/). The resulting annotation includes 40,821 transcribed mRNAs from 13,746 protein-coding and 5,678 non-coding genes.

## Methods

### Sample acquisition and nucleic acid extraction

A female *Bombus pratorum* (iyBomPrat1) was collected from Wytham Woods, Oxfordshire (biological vice-county: Berkshire), UK (latitude 51.77, longitude –1.33) on 20 August 2019. The specimen was taken from woodland habitat by Liam Crowley (University of Oxford) by netting. The specimen was identified by the collector and snap-frozen on dry ice. This specimen was used for genome sequencing and Hi-C scaffolding.

A second female *B. pratorum* specimen (iyBomPrat2) was used for RNA sequencing. The iyBomPrat2 specimen was collected by Olga Sivell (Natural History Museum) from woodland edge in Luton, UK (latitude 51.88, longitude –0.37) on 6 May 2020. The specimen was identified by Duncan Sivell (Natural History Museum) and snap-frozen on dry ice.

DNA was extracted at the Tree of Life laboratory, Wellcome Sanger Institute (WSI). The iyBomPrat1 sample was weighed and dissected on dry ice with abdomen tissue set aside for Hi-C sequencing. Head and thorax tissue was disrupted using a Nippi Powermasher fitted with a BioMasher pestle. High molecular weight (HMW) DNA was extracted using the Qiagen MagAttract HMW DNA extraction kit. Low molecular weight DNA was removed from a 20 ng aliquot of extracted DNA using the 0.8X AMpure XP purification kit prior to 10X Chromium sequencing; a minimum of 50 ng DNA was submitted for 10X sequencing. HMW DNA was sheared into an average fragment size of 12–20 kb in a Megaruptor 3 system with speed setting 30. Sheared DNA was purified by solid-phase reversible immobilisation using AMPure PB beads with a 1.8X ratio of beads to sample to remove the shorter fragments and concentrate the DNA sample. The concentration of the sheared and purified DNA was assessed using a Nanodrop spectrophotometer and Qubit Fluorometer and Qubit dsDNA High Sensitivity Assay kit. Fragment size distribution was evaluated by running the sample on the FemtoPulse system.

RNA was extracted from head tissue of iyBomPrat2 in the Tree of Life Laboratory at the WSI using TRIzol, according to the manufacturer’s instructions. RNA was then eluted in 50 μl RNAse-free water and its concentration assessed using a Nanodrop spectrophotometer and Qubit Fluorometer using the Qubit RNA Broad-Range (BR) Assay kit. Analysis of the integrity of the RNA was done using Agilent RNA 6000 Pico Kit and Eukaryotic Total RNA assay.

### Sequencing

Pacific Biosciences HiFi circular consensus and 10X Genomics read cloud DNA sequencing libraries were constructed according to the manufacturers’ instructions. Poly(A) RNA-Seq libraries were constructed using the NEB Ultra II RNA Library Prep kit. DNA and RNA sequencing were performed by the Scientific Operations core at the WSI on Pacific Biosciences SEQUEL II (HiFi), Illumina HiSeq 4000 (RNA-Seq) and HiSeq X Ten (10X) instruments. Hi-C data were also generated from abdomen tissue of iyBomPrat1 using the Arima v2 kit and sequenced on the HiSeq X Ten instrument.

### Genome assembly, curation and evaluation

Assembly was carried out with Hifiasm ( Cheng *et al.*, 2021) and haplotypic duplication was identified and removed with purge_dups ( Guan *et al.*, 2020). One round of polishing was performed by aligning 10X Genomics read data to the assembly with Long Ranger ALIGN, calling variants with FreeBayes ( Garrison & Marth, 2012). The assembly was then scaffolded with Hi-C data ( Rao *et al.*, 2014) using SALSA2 ( Ghurye *et al.*, 2019). The assembly was checked for contamination and corrected using the gEVAL system ( Chow *et al.*, 2016) as described previously ( Howe *et al.*, 2021). Manual curation was performed using gEVAL, HiGlass ( Kerpedjiev *et al.*, 2018) and Pretext ( Harry, 2022). The mitochondrial genome was assembled using MitoHiFi ( Uliano-Silva *et al.*, 2022), which performed annotation using MitoFinder ( Allio *et al.*, 2020). To evaluate the assembly, MerquryFK was used to estimate consensus quality (QV) scores and *k*-mer completeness ( Rhie *et al.*, 2020). The genome was analysed and BUSCO scores ( Manni *et al.*, 2021; Simão *et al.*, 2015) were calculated within the BlobToolKit environment ( Challis *et al.*, 2020). Table 3 contains a list of software tool versions and sources.

**Table 3.** Software tools: versions and sources.

Software tool	Version	Source
BlobToolKit	4.0.7	https://github.com/blobtoolkit/blobtoolkit
BUSCO	5.3.2	https://gitlab.com/ezlab/busco
FreeBayes	1.3.1-17-gaa2ace8	https://github.com/freebayes/freebayes
gEVAL	N/A	https://geval.org.uk/
Hifiasm	0.12	https://github.com/chhylp123/hifiasm
HiGlass	1.11.6	https://github.com/higlass/higlass
Long Ranger ALIGN	2.2.2	https://support.10xgenomics.com/genome-exome/software/pipelines/latest/advanced/other-pipelines
Merqury	MerquryFK	https://github.com/thegenemyers/MERQURY.FK
MitoHiFi	2	https://github.com/marcelauliano/MitoHiFi
PretextView	0.2	https://github.com/wtsi-hpag/PretextView
purge_dups	1.2.3	https://github.com/dfguan/purge_dups
SALSA	2.2	https://github.com/salsa-rs/salsa

### Genome annotation

The Ensembl gene annotation system ( Aken *et al.*, 2016) was used to generate annotation for the *Bombus pratorum* assembly (GCA_930367275.1). Annotation was created primarily through alignment of transcriptomic data to the genome, with gap filling via protein-to-genome alignments of a select set of proteins from UniProt ( UniProt Consortium, 2019).

### Ethics and compliance issues

The materials that have contributed to this genome note have been supplied by a Darwin Tree of Life Partner. The submission of materials by a Darwin Tree of Life Partner is subject to the Darwin Tree of Life Project Sampling Code of Practice. By agreeing with and signing up to the Sampling Code of Practice, the Darwin Tree of Life Partner agrees they will meet the legal and ethical requirements and standards set out within this document in respect of all samples acquired for, and supplied to, the Darwin Tree of Life Project. All efforts are undertaken to minimise the suffering of animals used for sequencing. Each transfer of samples is further undertaken according to a Research Collaboration Agreement or Material Transfer Agreement entered into by the Darwin Tree of Life Partner, Genome Research Limited (operating as the Wellcome Sanger Institute), and in some circumstances other Darwin Tree of Life collaborators.

## Data Availability

European Nucleotide Archive: *Bombus pratorum* (early bumblebee). Accession number PRJEB48116; https://identifiers.org/ena.embl/PRJEB48116. ( Wellcome Sanger Institute, 2022) The genome sequence is released openly for reuse. The *Bombus pratorum* genome sequencing initiative is part of the Darwin Tree of Life (DToL) project. All raw sequence data and the assembly have been deposited in INSDC databases. [ *If genome not annotated:* The genome will be annotated using available RNA-Seq data and presented through the Ensembl pipeline at the European Bioinformatics Institute.] Raw data and assembly accession identifiers are reported in Table 1.

## Author information

Members of the University of Oxford and Wytham Woods Genome Acquisition Lab are listed here: https://doi.org/10.5281/zenodo.4789928.

Members of the Natural History Museum Genome Acquisition Lab are listed here: https://doi.org/10.5281/zenodo.4790042.

Members of the Darwin Tree of Life Barcoding collective are listed here: https://doi.org/10.5281/zenodo.4893703.

Members of the Wellcome Sanger Institute Tree of Life programme are listed here: https://doi.org/10.5281/zenodo.4783585.

Members of Wellcome Sanger Institute Scientific Operations: DNA Pipelines collective are listed here: https://doi.org/10.5281/zenodo.4790455.

Members of the Tree of Life Core Informatics collective are listed here: https://doi.org/10.5281/zenodo.5013541.

Members of the Darwin Tree of Life Consortium are listed here: https://doi.org/10.5281/zenodo.4783558.

## References

[ref-1] Aken BL Ayling S Barrell D : The Ensembl gene annotation system. *Database (Oxford).* 2016;2016:baw093. 10.1093/database/baw093 27337980 PMC4919035

[ref-2] Alford DV : A study of the hibernation of bumblebees (Hymenoptera: Bombidae) in southern England. *J Anim Ecol.* 1969;149–170. 10.2307/2743

[ref-3] Allio R Schomaker-Bastos A Romiguier J : MitoFinder: Efficient automated large‐scale extraction of mitogenomic data in target enrichment phylogenomics. *Mol Ecol Resour.* 2020;20(4):892–905. 10.1111/1755-0998.13160 32243090 PMC7497042

[ref-4] Benton T : Chapter 9: The British Species, in *Bumblebees* . London, UK: HarperCollins Publishers,2006;338–342.

[ref-5] Challis R Richards E Rajan J : BlobToolKit - interactive quality assessment of genome assemblies. *G3 (Bethesda).* 2020;10(4):1361–1374. 10.1534/g3.119.400908 32071071 PMC7144090

[ref-6] Cheng H Concepcion GT Feng X : Haplotype-resolved *de novo* assembly using phased assembly graphs with hifiasm. *Nat Methods.* 2021;18(2):170–175. 10.1038/s41592-020-01056-5 33526886 PMC7961889

[ref-7] Chow W Brugger K Caccamo M : gEVAL — a web-based browser for evaluating genome assemblies. *Bioinformatics.* 2016;32(16):2508–2510. 10.1093/bioinformatics/btw159 27153597 PMC4978925

[ref-8] Edwards M Jenner M : Field guide to the bumblebees of Great Britain & Ireland. Eastbourne: Ocelli Ltd,2005. Reference Source

[ref-9] Foster G Bennett J Sparks T : An assessment of bumblebee ( *Bombus* spp) land use and floral preference in UK gardens and allotments cultivated for food. *Urban Ecosyst.* 2017;20(2):425–434. 10.1007/s11252-016-0604-7

[ref-10] Garrison E Marth G : Haplotype-based variant detection from short-read sequencing.2012. 10.48550/arXiv.1207.3907

[ref-11] Ghurye J Rhie A Walenz BP : Integrating Hi-C links with assembly graphs for chromosome-scale assembly. *PLoS Comput Biol.* 2019;15(8):e1007273. 10.1371/journal.pcbi.1007273 31433799 PMC6719893

[ref-12] Goulson D Darvill B : Niche overlap and diet breadth in bumblebees; are rare species more specialized in their choice of flowers? *Apidologie.* 2004;35(1):55–63. 10.1051/apido:2003062

[ref-13] Guan D McCarthy SA Wood J : Identifying and removing haplotypic duplication in primary genome assemblies. *Bioinformatics.* 2020;36(9):2896–2898. 10.1093/bioinformatics/btaa025 31971576 PMC7203741

[ref-14] Harry E : PretextView (Paired REad TEXTure Viewer): A desktop application for viewing pretext contact maps.2022; (Accessed: 19 October 2022). Reference Source

[ref-15] Howe K Chow W Collins J : Significantly improving the quality of genome assemblies through curation. *GigaScience.* Oxford University Press.2021;10(1):giaa153. 10.1093/gigascience/giaa153 33420778 PMC7794651

[ref-16] Kerpedjiev P Abdennur N Lekschas F : HiGlass: Web-based visual exploration and analysis of genome interaction maps. *Genome Biol.* 2018;19(1):125. 10.1186/s13059-018-1486-1 30143029 PMC6109259

[ref-17] Knight ME Martin AP Bishop S : An interspecific comparison of foraging range and nest density of four bumblebee ( *Bombus*) species. *Mol Ecol.* 2005;14(6):1811–1820. 10.1111/j.1365-294X.2005.02540.x 15836652

[ref-18] Lye GC Osborne JL Park KJ : Using citizen science to monitor *Bombus* populations in the UK: nesting ecology and relative abundance in the urban environment. *J Insect Conserv.* 2012;16(5):697–707. 10.1007/s10841-011-9450-3

[ref-19] Manni M Berkeley MR Seppey M : BUSCO Update: Novel and Streamlined Workflows along with Broader and Deeper Phylogenetic Coverage for Scoring of Eukaryotic, Prokaryotic, and Viral Genomes. *Mol Biol Evol.* 2021;38(10):4647–4654. 10.1093/molbev/msab199 34320186 PMC8476166

[ref-20] Prŷs-Jones OE Corbet SA : Bumblebees.New York: Cambridge University Press.1987.

[ref-21] Rao SSP Huntley MH Durand NC : A 3D map of the human genome at kilobase resolution reveals principles of chromatin looping. *Cell.* 2014;159(7):1665–1680. 10.1016/j.cell.2014.11.021 25497547 PMC5635824

[ref-22] Rhie A Walenz BP Koren S : Merqury: Reference-free quality, completeness, and phasing assessment for genome assemblies. *Genome Biol.* 2020;21(1):245. 10.1186/s13059-020-02134-9 32928274 PMC7488777

[ref-23] Rhie A McCarthy SA Fedrigo O : Towards complete and error-free genome assemblies of all vertebrate species. *Nature.* 2021;592(7856):737–746. 10.1038/s41586-021-03451-0 33911273 PMC8081667

[ref-24] Simão FA Waterhouse RM Ioannidis P : BUSCO: assessing genome assembly and annotation completeness with single-copy orthologs. *Bioinformatics.* 2015;31(19):3210–3212. 10.1093/bioinformatics/btv351 26059717

[ref-25] Uliano-Silva M Ferreira JGRN Krasheninnikova K : MitoHiFi: a python pipeline for mitochondrial genome assembly from PacBio High Fidelity reads. *bioRxiv.* [Preprint].2022. 10.1101/2022.12.23.521667 PMC10354987 37464285

[ref-26] UniProt Consortium: UniProt: a worldwide hub of protein knowledge. *Nucleic Acids Res.* 2019;47(D1):D506–D515. 10.1093/nar/gky1049 30395287 PMC6323992

[ref-27] Wellcome Sanger Institute: The genome sequence of the Early Bumblebee, *Bombus pratorum* (Linnaeus, 1761). European Nucleotide Archive.[dataset], accession number PRJEB48116,2022.

