# Supplementary material for: The genome sequence of the Early Bumblebee, Bombus pratorum (Linnaeus, 1761)
Source: Wellcome Open Res. 2023 Mar 29;8:143. [Version 1] doi: 10.12688/wellcomeopenres.19250.1 (PMC10632595; doi:10.12688/wellcomeopenres.19250.1)
Supplement: Peer review history

# Reviewer report: Reviewer response for version 1

**DOI:** 10.21956/wellcomeopenres.21333.r69183

**Published:** 2023-11-08

**Authors:** Rodolpho S. T. Menezes

**Affiliations:** 1 Department of Biological Sciences, State University of Santa Cruz, Ilhéus, Brazil

This genome report describes the genome sequencing of the early bumblebee, *Bombus pratorum*. This genome will be interesting for comparative genomics investigation within Apidae and also for evolution and conservation studies. Moreover, the manuscript is well written. Below I list few comments to the authors to improve the manuscript:

- Genome assembly: Since you assembled mitochondrial genome I think it is interesting you show general (GC/AT content…) mitochondrial information about this species.
- Table 3: Please include MitoFinder information.

Are sufficient details of methods and materials provided to allow replication by others?

Yes

Is the rationale for creating the dataset(s) clearly described?

Yes

Are the datasets clearly presented in a useable and accessible format?

Yes

Are the protocols appropriate and is the work technically sound?

Yes

Reviewer Expertise:

Cytogenetics, Phylogenomics, and Phylogeography.

I confirm that I have read this submission and believe that I have an appropriate level of expertise to confirm that it is of an acceptable scientific standard.

# Reviewer report: Reviewer response for version 1

**DOI:** 10.21956/wellcomeopenres.21333.r69176

**Published:** 2023-10-25

**Authors:** Cheng Sun

**Affiliations:** 1 College of Life Sciences, Capital Normal University, Beijing, Beijing, China

This study provided a high-quality of genome assembly for bumblebee *Bombus pratorum, *with workers of the first brood appearing from as early as February. This genome sequence will enrich the current bumblebee genomic resources and facilitate studies on the diversification and adaptation of bumblebees.

 This manuscript was well-written. The Methods are sound and the Results are reliable.

Are sufficient details of methods and materials provided to allow replication by others?

Yes

Is the rationale for creating the dataset(s) clearly described?

Yes

Are the datasets clearly presented in a useable and accessible format?

Yes

Are the protocols appropriate and is the work technically sound?

Yes

Reviewer Expertise:

Bumblebee genome evolution

I confirm that I have read this submission and believe that I have an appropriate level of expertise to confirm that it is of an acceptable scientific standard.
